# A geometric model for the human pulmonary valve in its fully open case

**DOI:** 10.1371/journal.pone.0199390

**Published:** 2018-06-25

**Authors:** Xiaoqin Shen, Lin Bai, Li Cai, Xiaoshan Cao

**Affiliations:** 1 School of Sciences, Xi’an University of Technology, Xi’an, 710054, P.R.China; 2 NPU-UoG International Cooperative Lab for Computation & Application in Cardiology, Northwestern Polytechnical University, Xi’an, 710072, P.R.China; 3 State Key Laboratory of Transducer Technology, Chinese Academy of Sciences, Shanghai, 200050, P.R.China; Worcester Polytechnic Institute, UNITED STATES

## Abstract

The human pulmonary valve, one of the key cardiac structures, plays an important role in circulatory system. However, there are few mathematical models to accurately simulate it. In this paper, we establish a geometric model of the normal human pulmonary valve from a mathematical perspective in the fully opening case. Based on the statistical data of the human pulmonary valves, we assume that the motions of the three cusps are symmetrical in the cardiac cycle. Thus, we first propose that each cusp is a part of the cylindrical shell according to its structure and physiological feature. The parameters for the pulmonary valve cusps in three-dimensional space are obtained by the fitting functions. We verify the accuracy of our results by comparing the areas of the pulmonary valve and pulmonary valve flap.

## Introduction

The heart is one of the most complicated and important organs. It consists of two atriums receiving blood flowing back to the heart and two ventricles pushing blood from the heart into the arteries. The blood moves through four valves: the mitral and tricuspid valves, through which blood flows from the atria to the ventricles, and the aortic and pulmonary valves, through which blood flows out of the ventricles. The main function of heart valves is to make sure that blood flows in the proper direction. The heartbeat known to us all is due to the opening and closing of the valves, and because our hearts beat about 100,000 times a day, over the average human lifetime of 79 years, our heart valves open and close almost 3 billion times. The average human heart rate is 72 beats per minute, and about 70 mL of blood flows through the heart per beat, so about 5 L of blood flows through the valves per minute. Each heart valve has its own distinguishing characteristics and performs its own unique functions.

**Fig 1:**
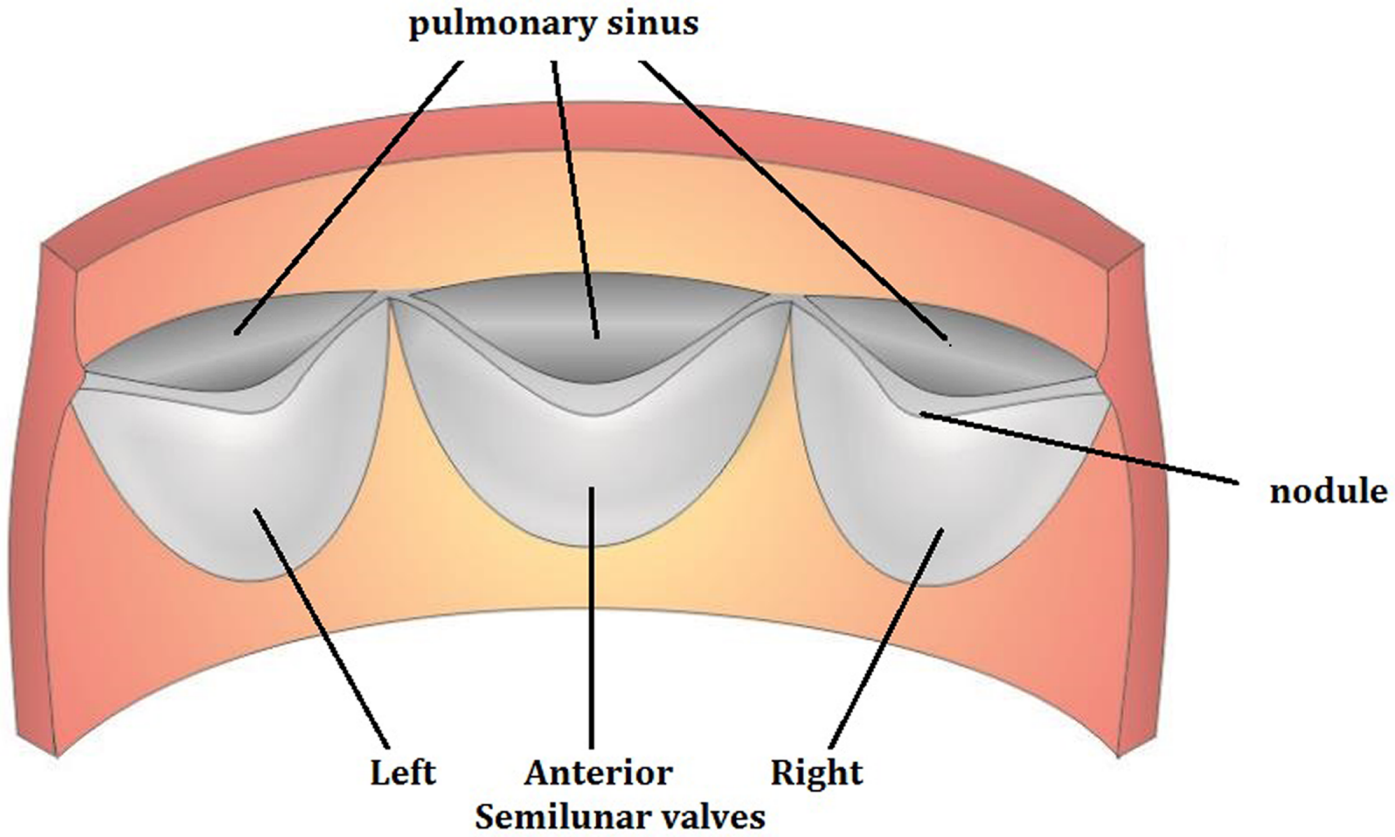
Posterior view of the human pulmonary valve (cf. [3]).

The pulmonary valve ensures that blood flows from the ventriculus dexter into pulmonary artery without allowing a significant reflux of blood flowing in the reverse direction. The pulmonary artery takes oxygen-depleted blood to the lung to absorb oxygen and release carbon dioxide. The pulmonary valve has three leaflets and a valve ring [1, 2], which is connected to the right ventricular funnel muscle. The three leaflets are called the anterior semilunar valve, left semilunar valve and right semilunar valve (Fig 1, cf. [3]). The anterior semilunar valve is linked to the right ventricular free wall, the left semilunar valve is linked to the septal band of the funnel wall, and the right semilunar valve is linked to every wall beam of the funnel wall. Each valve is attached to the free margin of the ventricular flange and extends to the free edge of the annular fibrosis. There is a pulmonary sinus between each semilunar valve and the pulmonary artery wall. When the pulmonary valve opens or closes, the pouches fill with blood, and when the blood is squeezed out, it can only flow from the ventriculus dexter to pulmonary artery.

In fact, a series of lesions may occur in the pulmonary valve that can have some effects on the human life. For example, the congenital pulmonary valve stenosis caused or accompanied the infundibular stenosis, the pulmonary incompetence give rise to the right-sided heart failure. Based on the fact that the pulmonary valve plays an important role in its own environment and it may appear a series of lesions. These makes the study of normal human heart pulmonary valves is of great significance, but there have been fewer studies of the pulmonary valve than of other heart valves. Most of the research has been related to the relationship between the pulmonary valve and human diseases, but some scholars have begun to study normal human normal pulmonary valves.

Maron and Hutchins [4] studied the development of human semilunar valves, including in embryos before the appearance of cellular semilunar valves and hearts but after the development of the mature fibroelastic valvular structure, which occurs around the time of birth. Stradins and others [5] concluded that the aortic and the pulmonary valves have similar structural elements and architecture, and that the pulmonary valve can be considered mechanically and structurally suitable for use as a replacement for the aortic valve. In 1967, Ross [6] presented the Ross program where the aortic valve is replaced by pulmonary valve. In 1997, Li *et al*. [7] studied the mechanical properties of aortic and pulmonary valves and showed that there are significant differences between the aortic and pulmonary valves. Stamm [8] did a survey of pulmonary valves in 1998. Soares *et al*. [9] studied the mechanical differences between the aortic valve and the pulmonary valve and the possible remodeling of the pulmonary valve in the aortic position. In 2009, Joyce and his team [10] verified the accuracy of this conclusion. Grbic *et al*. [11] proposed a complete and modular patient-specific model of the cardiac valvular apparatus based on 4D cardiac computed tomography data. The Saremi team [12] focused on the role of CT and MR imaging in assessing morphology, function and developmental malformations of pulmonary valves. In 2014, Sun *et al*. [13] completed the geometrical reconstruction of the valve, the modeling of tissue characteristics and the heart valve function as well as intervention of the aortic valve and the mitral valve. Naso *et al*. [14] developed a new soft-tissue analysis method for *α*-Gal quantification in xenograft heart valves before and after the detergent-based or equivalent cell removal procedure. Fan *et al*. [15] have presented an approach for optimal leaflet shape design based on finite element simulation of a mechanically anisotropic, elastomeric scaffold for PV replacement is presented. In 2017, Li *et al*. [16] established a multi-scale electrokinetics model of the left ventricle to simulate calcium dynamics in individual myocytes.

It is necessary to do more research in both medicine and mathematics, especially completing the approximate reproduction of the pulmonary valve, it provide theoretical support for the clinical medicine on the construction of artificial biological heart valves and the treatment of heart valve-related injuries. In this paper, we describe our studies on the research of the normal human pulmonary valves. The conclusions, which are based on fundamental analysis, include a description of the geometrical shape of pulmonary valves and geometry parameter expressions that relate to them. The main components of our approach are the following: (1) We assume that the motion in a cardiac cycle of each cusp is the same and the motion is symmetrical, so we only study a single cusp. (2) We assume that each pulmonary valve cusp functions as a part of cylindrical elastic shells according to its structure and physiological feature. (3) Based on medical its structure and relevant data, we use the fitting function to determine the relevant parameters. (4) We use other data from the pulmonary valve verified the accuracy of our results.

## Methods and results

### The realization of the basic model

**Fig 2:**
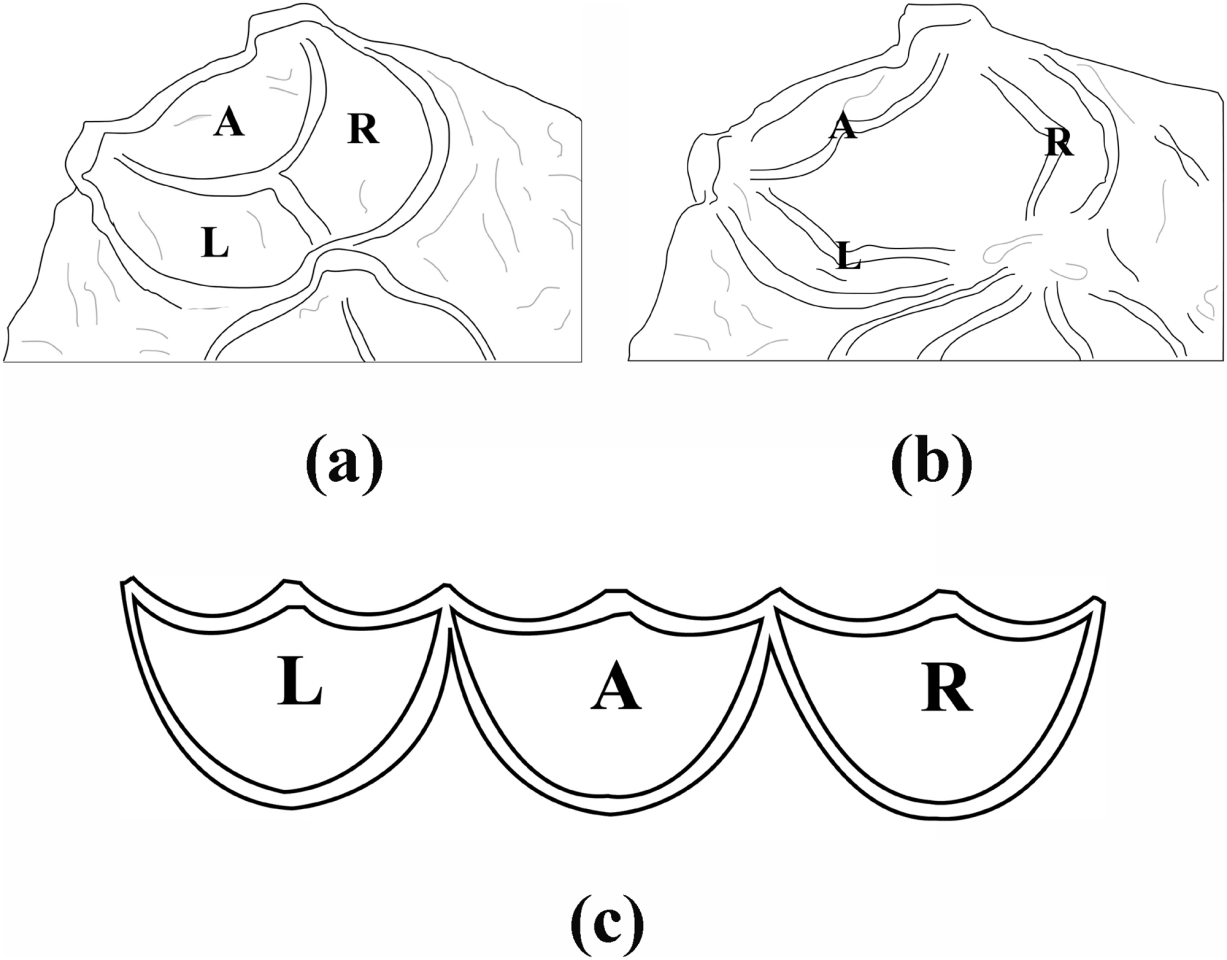
A schematic plot of the human pulmonary valve. (a) is the pulmonary valve during diastole (cf. [15]) and (b) is the pulmonary valve during systole (cf. [18]) (c) is the expanded semilunar valve. A: anterior leaflet, L: left leaflet, R: right leaflet.

The intersection of the cusps of the pulmonary valve of the adult heart is Y-shaped when the pulmonary valve is closed, but when the valve is open, the opening takes on a circular shape [17]. Fig 2 shows a schematic plot of the pulmonary valve of the human body, where (a) and (b) are from [15] and [18], (c) is plotted by ourselves. The research described in this paper is based on the following assumptions: (1) We assume that the three cusps have the same structure, so they take on a completely symmetrical shape with an angle between the lines that bisect the cusps of 120°. (2) We can focus our attention on one individual cusp, disregarding how the cusps combine. (3) We assume that the free edge has even structures with smooth edges. We focus our research on the right semilunar pulmonary valve.

Based on the structure of the pulmonary valve and the description of the physiological features, we will assume the pulmonary valve can be described as a cylindrical shell within an elastic shell.

### The geometry and design parameters of the cusps

The goal of this section is to confirm the geometrical shape of the natural pulmonary valve and describe its parametric expression.

Table 1 shows the literature values for the widths and the heights of the cusps of the pulmonary valve [19]. The height refers to the distance from the free edge of the pulmonary valve to its attachment midpoint, and the width refers to the distance between the pedicles.

**Table 1:** The width and height in millimeters of the pulmonary valve cusps.

Width and Height	Width(mm)	Height(mm)
the left cusp	21.61 ± 3.56	11.78 ± 2.36
the right cusp	21.58 ± 4.25	12.03 ± 2.38
the anterior cusp	21.41 ± 4.14	12.05 ± 2.40

**Fig 3:**
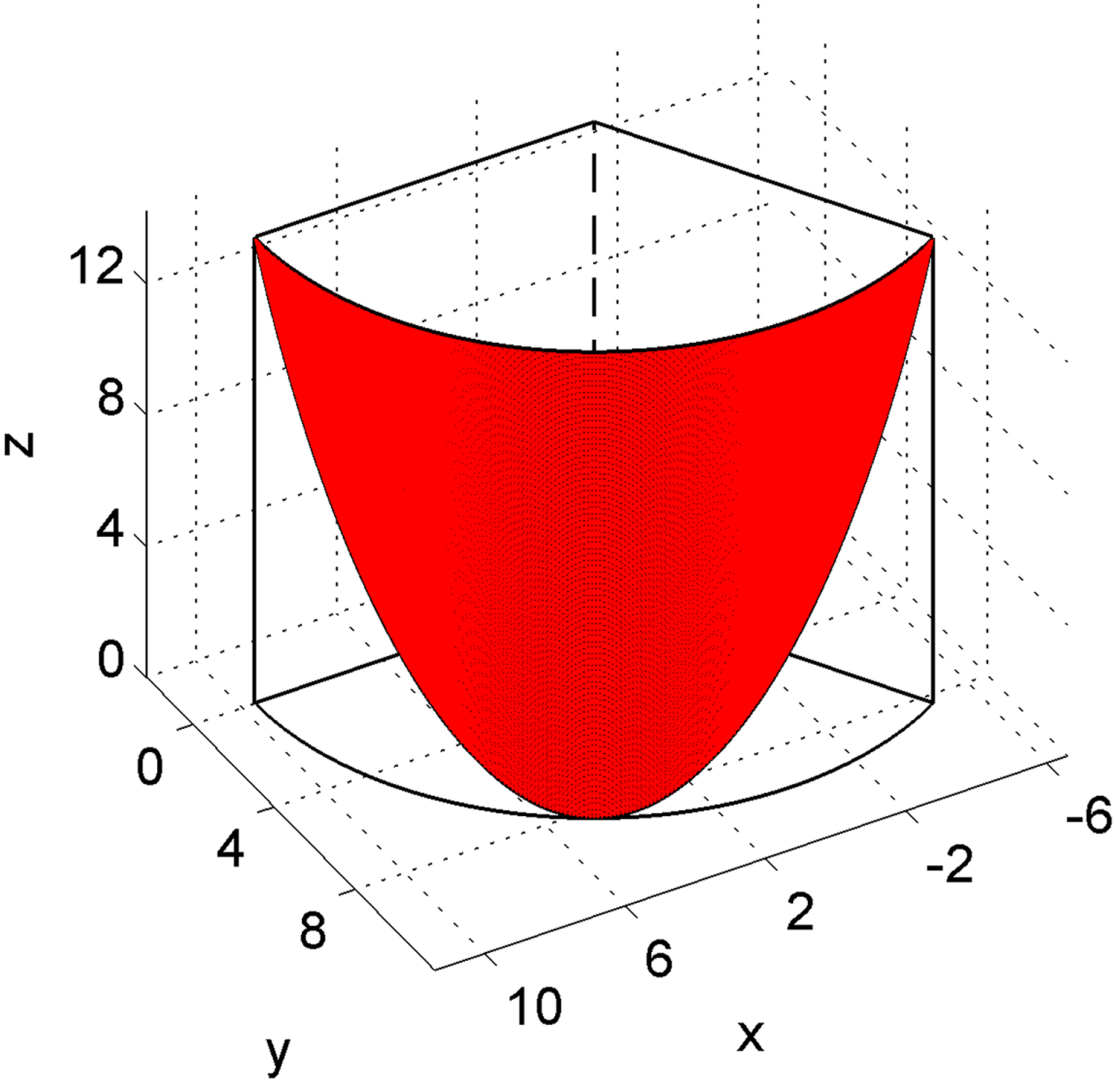
The model for the geometrical shape of a cusp in the pulmonary valve. The leaflets is considered to be one-third of the cylindrical shell.

**Fig 4:**
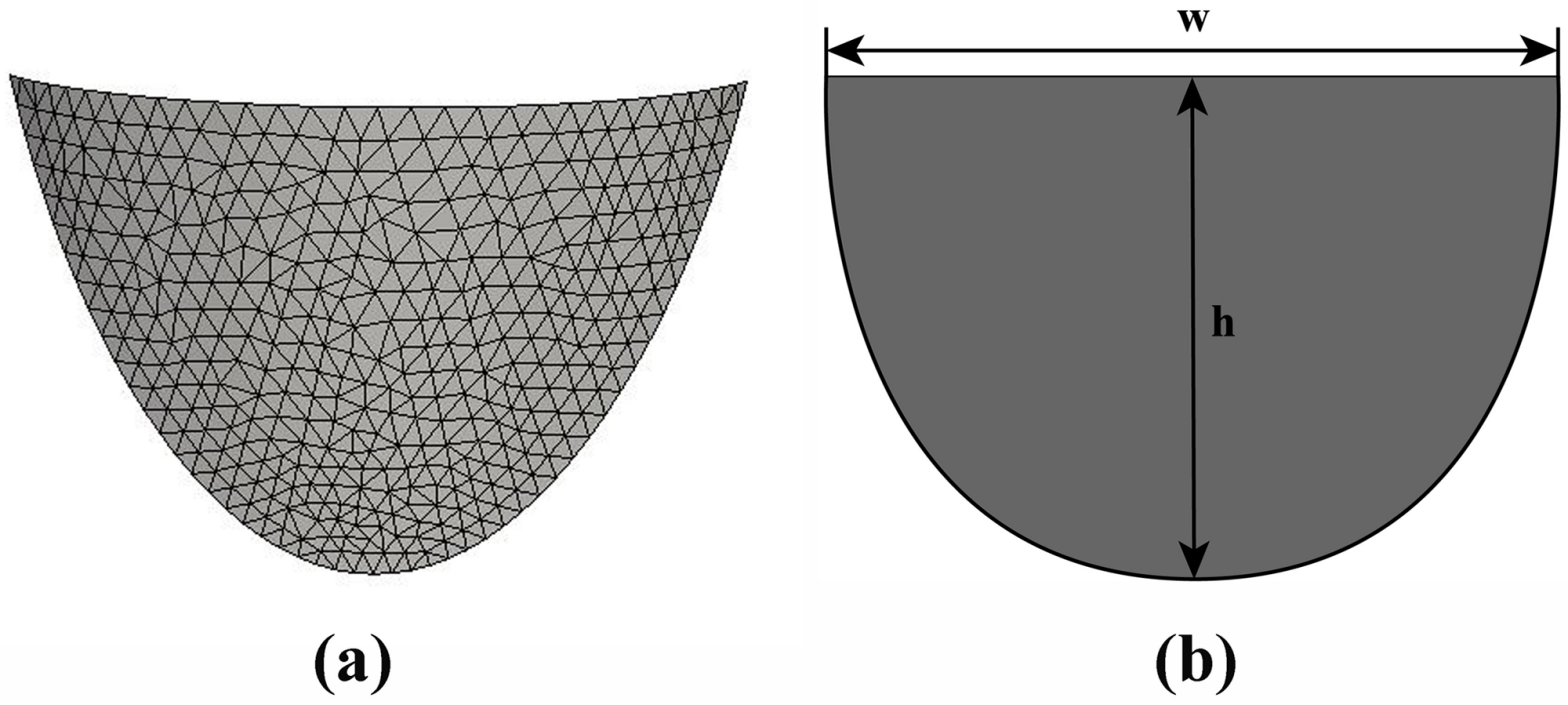
(a)The leaflet shape shown in 3D (b)The leaflet shape shown “laid flat” in 2D.

The right semilunar valve is deemed as a portion of the cylinder shell [20] which is cut along the major axis of the xy plane (Fig 3). We have to determine the valve of every coefficient including *r* and *h*, and the range of every variable including *θ* and *z* (see Appendix), to fit the shape of the right valve. In fact, the size of the pulmonary valves is different based on individual variation, so we give an approximate value, that is take the width of the semilunar valve as 20.211mm and the maximum height of the semilunar valve as 14.20mm (Fig 4). Based on the design of the blade parameters as a part of the cylindrical shells (Fig 3), we derived *r* = 9.65mm as the radius of cut round and *h* = 14.20mm as the maximum height of the leaflet, the range of the parameter *θ* was assumed to be $\left[0,\frac{2\pi }{3}\right]$ because the semilunar valve is one-third of a cylindrical shell. The parameter *z* is a function of the variable *θ*, and it determines the shape of the semilunar valve.

**Fig 5:**
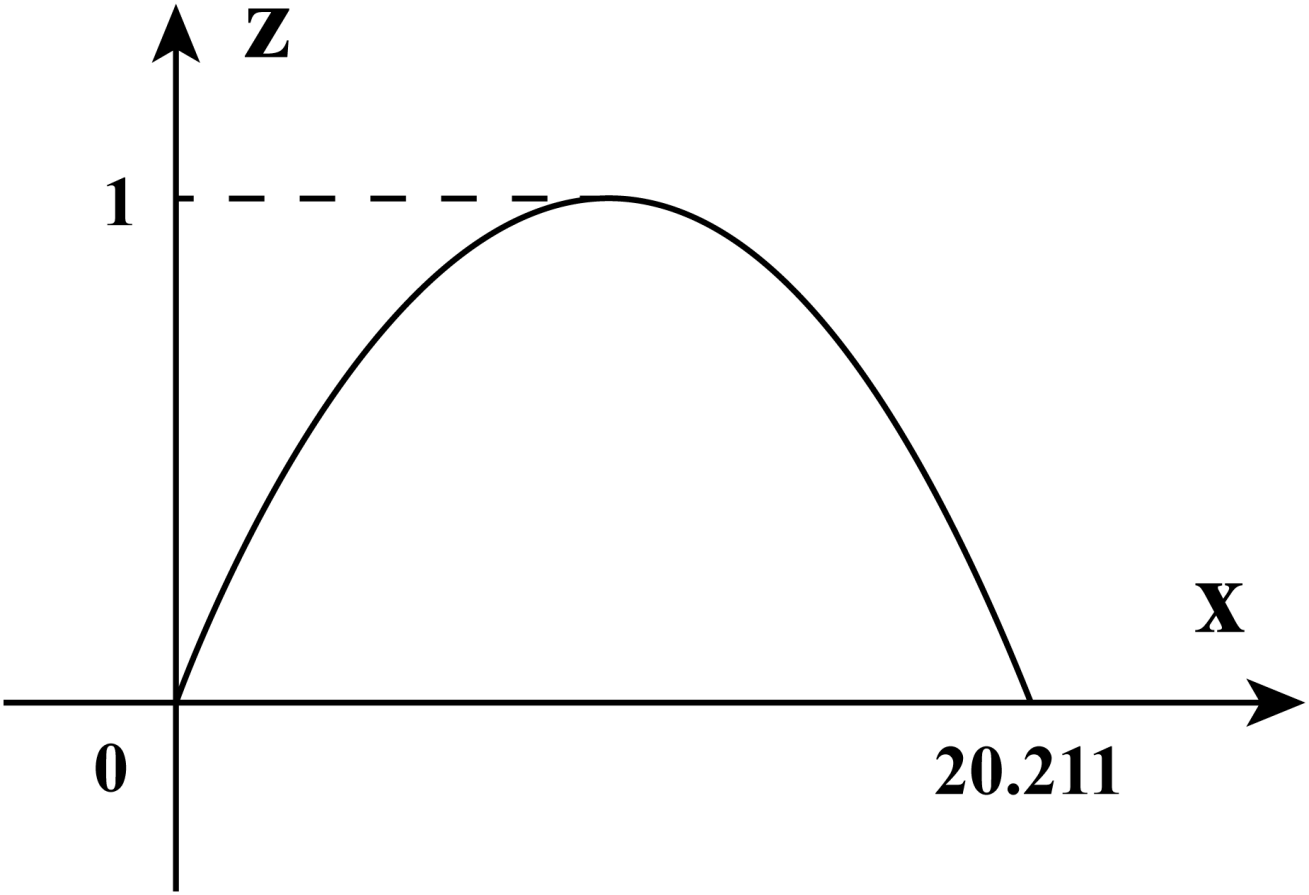
The boundary curve of the semilunar valve.

In order to determine the range of the *z*, we have prepared a sketch map of the right leaflet boundary in a plane rectangular coordinate system (Fig 5). We will study the upper boundary of the schematic diagram 5 and expect to get the result of variable *z*.

Based on the height and width of leaflet as well as the shape of the semilunar valve, we choose the sine function to simulate the upper boundary of the semilunar valve because the sine function introduces relatively few variables and provides good smoothness, we set its peak as (10.1055, 1) and made its lowest point as (0, 0), (20.211, 0). Then we did the function fitting and obtained the function expression of the upper boundary as $z=\sin(\frac{\pi }{20.211}x)$, where *x* represents the width of the semilunar valve and *x* ∈ [0, 20.211]. This is the result of our fitting about the upper boundary, whereas our aim is complete the approximate reproduction of the valve in a three-dimensional space, thus we look for the relationship between the width *x* of the valve and the variable *θ*. We notice that the width *x* is the arc length in the space where *r* is the radius and *θ* is the center of the circle. We use the expression *x* = *rθ* to obtain the function expression for *z* in a three-dimensional space $z=\sin(\frac{9650}{20211}\pi \theta )$.

Finally, by making use of the three geometric parameters, we obtained the function expression for the right semilunar valve as follows:

$$\begin{array}{c} \vec{\theta }\left(\theta ,z\right)=\left(9.65\cos\theta ,9.65\sin\theta ,14.2z\right), \end{array}$$ (1)

where $\theta \in [0,\frac{2\pi }{3}]$ and $z\in [0,\sin\left(\frac{9650}{20211}\pi \theta \right)]$.

### Validation

To guarantee the accuracy of the parametric equations described above, we calculated that the disc ring circumference is 60.6327mm and the area of the right cusp is 182.6220mm^2^, and the diameter of the annulus is 19.3mm. Therefore, the area of pulmonary valve is 547.8660mm^2^. According to the scientific literature [19], the ratio of the area of pulmonary valve flap to the area of pulmonary valve is 1: 1.55. Using this ratio, we calculated the area of pulmonary valve flap as 353.4619mm^2^. Table 2 shows the comparison of these values to those of Yang [19]. The fact that our values are close to the values found by Yang verifies the correctness of the parametric equations for the human pulmonary valve. Therefore, we can use our model to describe the pulmonary valve.

**Table 2:** Comparison of our values with Yang’s values [19].

Workers	Area of the right cusp valve(mm^2^)	Valve ring		Area of Valve orifice(mm^2^)
		diameter (mm)	perimeter (mm)	
Our results	182.6220	19.3	60.6327	353.4619
Yang’s [19]	238.71 ± 63.47	18.2 ± 3.2	57.177 ± 10.053	456.60 ± 106.62

### Coupling

**Fig 6:**
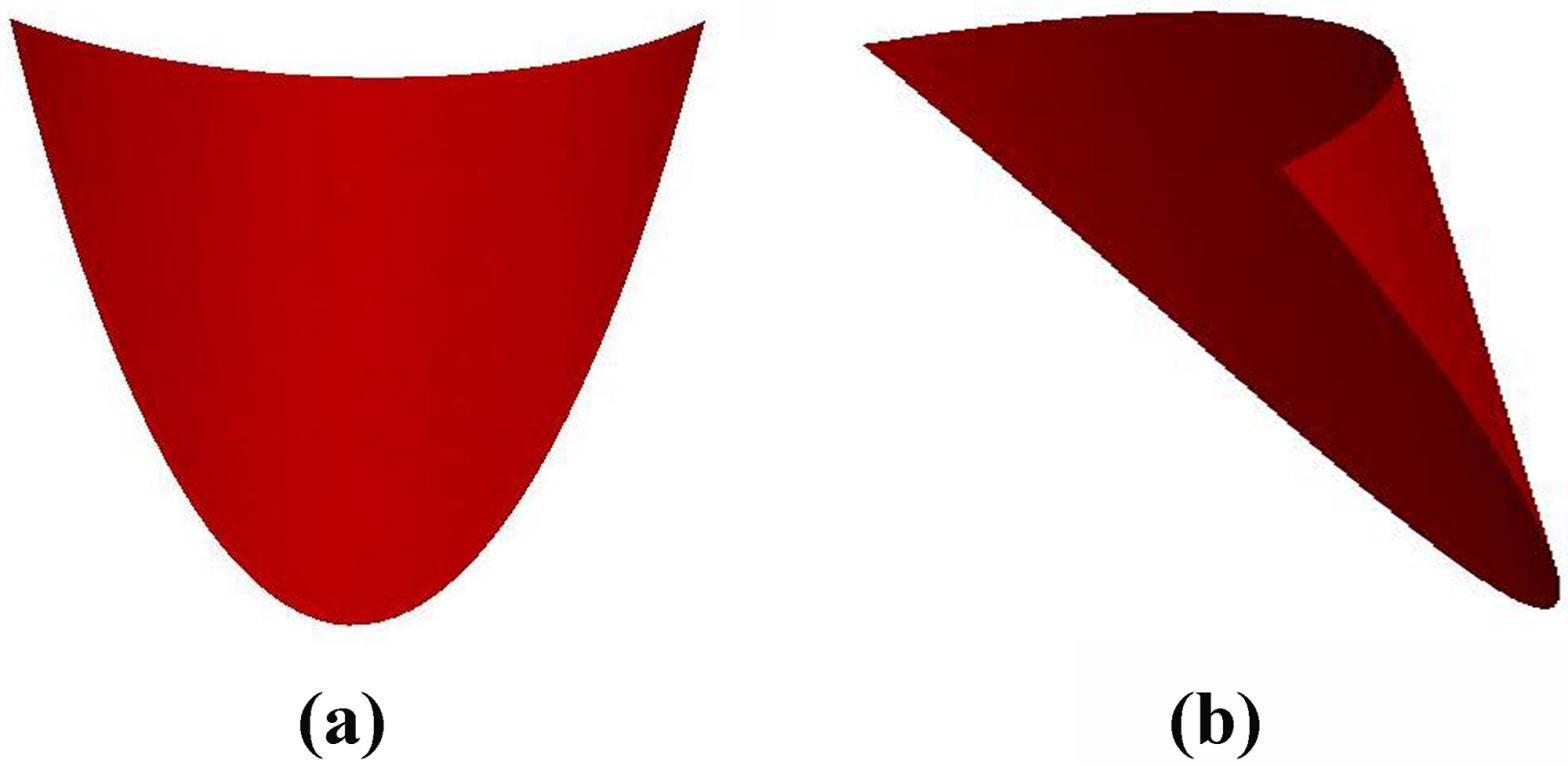
Visualization of the right half of the valve cusp in three-dimensional space. (a) is the image of the front view of a single cusp and (b) is the side view.

**Fig 7:**
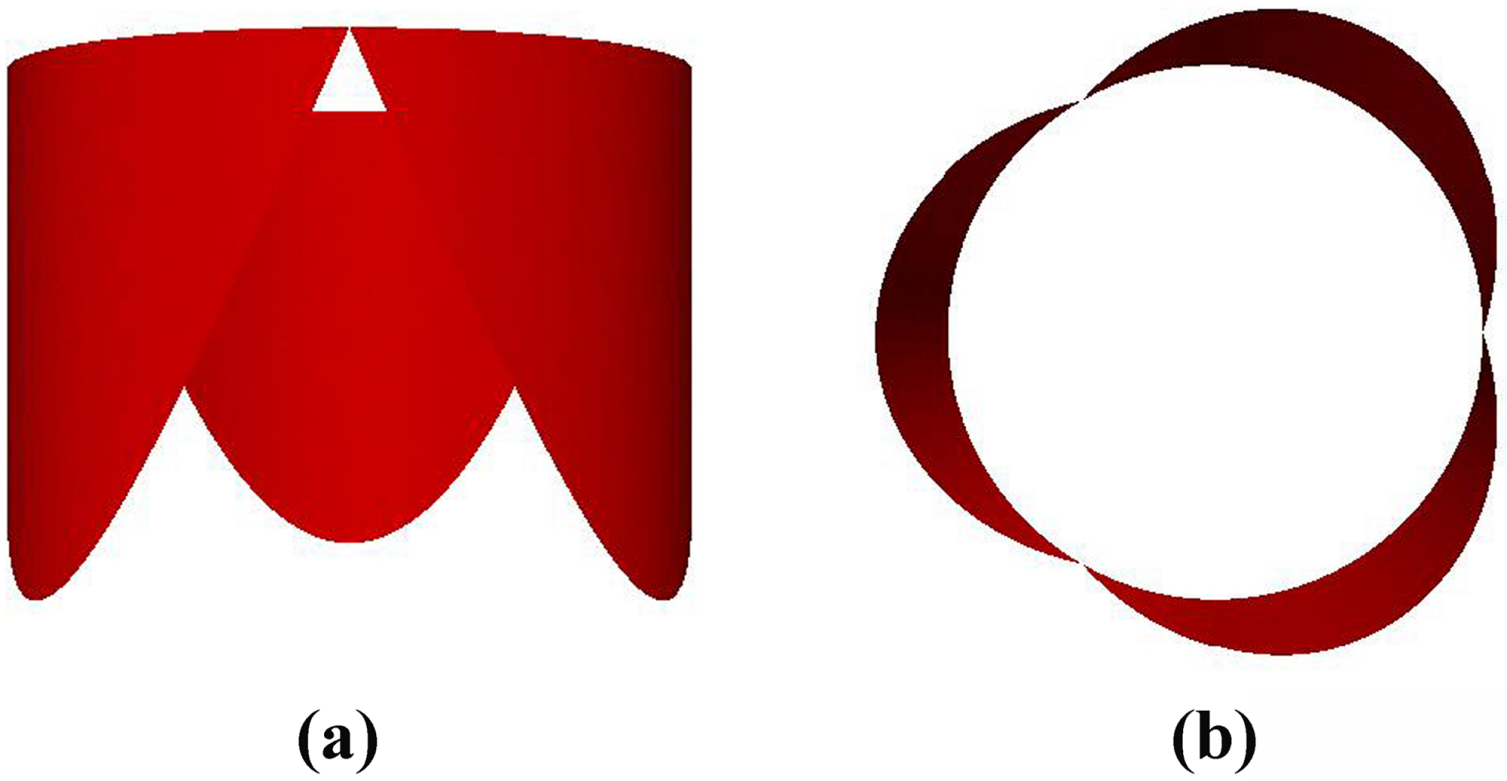
The combined graph of the human pulmonary valve cusps in three-dimensional space. (a) is the image observed from the front and (b) is the image observed from the top.

We have visualized the results of a function fitting of the human pulmonary valve leaflets, ie individual leaflet. Fig 6 shows the results of the function fitting for the right semilunar cusp. But we want to get the geometry of the entire pulmonary valve, according to the assumption that the three leaflets of our pulmonary valve are completely symmetrical, ideally, we can accomplish an approximate reproduction of the pulmonary valve by rotating a single valve, and finally we obtained a total graph of the human pulmonary valve from different angles. Fig 7(a) shows the side view, and Fig 7(b) shows the top view.

## Discussion

Our model is an attempt to achieve an approximate reproduction of the pulmonary valve by established a basic model. Some simplifications were made when we selected our model, our study ignored the height differences among the valve cusps, assumed the cusps are constant in average thickness, and assumed that their edges are smooth. But for real pulmonary valves, the cusps are not entirely symmetrical, the thicknesses vary and their edges are not absolutely smooth, especially when there is an occlusion of the pulmonary valve, it may be that an occlusion of the pulmonary valve distorts the lobular edge slightly, so the cusps do not overlap at the binding sites when the pulmonary valve is closed. Several improvements can be added to our model in the future for better accuracy and applicability.

Although most designs of heart valves focus on stress-strain analyses, here our main goal is to analyzed from relatively simple geometric shapes. In this paper, we presented a model of the geometric shape and parametric expression of the normal pulmonary valve of the human body. We assumed that the three cusps of the human pulmonary valve were symmetrical in their motion in a cardiac cycle and that we could describe each cusp as part of the cylindrical shell. Next, the corresponding parameter expressions of the pulmonary valve cusps in three-dimensional space were obtained by fitting functions. Finally, we verified the accuracy of our results by comparing our values for the key parameters to accepted literature values. We pay attention to parametric equations and geometric shapes as the primary metric makes the effort important. We will add material properties and introduce an external force field into our model. The improvements will be our future direction and we will take considerable effort to implement. We expect that our model to more accurately simulate the motion of the pulmonary valve over the complete cardiac cycle.

## Appendix

The parametric equation of the cylinder is given as follows:

$$\begin{array}{c} \vec{\theta }\left(\theta ,z\right)=\left(r\cos\theta ,r\sin\theta ,hz\right) \end{array}$$

where *θ* ∈ [0, 2*π*] and *z* ∈ [0, 1] are variables, *r* is the radius of cut round and *h* is the maximum height of cylindrical.

## Supporting information

S1 Fig The model for the geometrical shape of a cusp in the pulmonary valve. It was plotted by Matlab on the basis of the size of the pulmonary valve in Table 1, and we offered the source file “S1_Fig.m” for Fig 3.
(M) Click here for additional data file.

S2 Fig Visualization results. They were coded by FreeFem++ and visualized by Paraview, and we offered the FreeFem++ code file “S2_Fig.edp” for Fig 6.
(EDP)
